# Gabapentin-induced drug-seeking-like behavior: a potential role for the dopaminergic system

**DOI:** 10.1038/s41598-020-67318-6

**Published:** 2020-06-26

**Authors:** Yusuf S. Althobaiti, Amal Alghorabi, Fahad S. Alshehri, Bandar Baothman, Atiah H. Almalki, Hashem O. Alsaab, Walaa Alsanie, Ahmed Gaber, Hussam Almalki, Abdulrahman S. Alghamdi, Ahmad Basfer, Sultan Althobaiti, Ana Maria Gregio Hardy, Zahoor A. Shah

**Affiliations:** 1 Department of Pharmacology and Toxicology, College of Pharmacy, Taif University, Health Science Campus, Airport Road, Al Haweiah, PO Box 888, Taif, 21974 Saudi Arabia; 2 Addiction and Neuroscience Research Unit, Biomedical Sciences Research Center, Taif University, Taif, Saudi Arabia; 3 General Administration for Precursors and Laboratories, Ministry of Interior, General Directorate of Narcotics Control, Riyadh, Saudi Arabia; 4 Department of Pharmacology and Toxicology, College of Pharmacy, Umm Al-Qura University, Makkah, Saudi Arabia; 5 Deanship of Scientific Research, Taif University, Taif, Saudi Arabia; 6 Department of Pharmaceutical Chemistry, College of Pharmacy, Taif University, Taif, Saudi Arabia; 7 Department of Pharmaceutics and Pharmaceutical Technology, College of Pharmacy, Taif University, Taif, Saudi Arabia; 8 Department of Clinical Laboratories Science, Faculty of Applied Medical Sciences, Taif University, Taif, Saudi Arabia; 9 Department of Biology, Faculty of Science, Taif University, Taif, Saudi Arabia; 10 Department of Pharmaceutical Care, Directorate of Health Affairs, Ministry of Health, Taif, Saudi Arabia; 11 Department of Physiology and Pharmacology, College of Medicine and Life Sciences, University of Toledo, Toledo, OH USA; 12 Department of Medicinal and Biological Chemistry, College of Pharmacy and Pharmaceutical Sciences, University of Toledo, Toledo, OH USA

**Keywords:** Reward, Neuroscience

## Abstract

Drugs of abuse represent a growing public health crisis. Accumulating evidence indicates that gabapentin (GBP), a prescription drug, is prone to misuse, abuse, withdrawal, and dependence. Commonly, drugs of abuse modulate the dopaminergic system to induce addiction. In this study, we used the conditioned place preference (CPP) model to investigate the involvement of the dopamine 1 (D1) receptor on the reward and reinforcement behavior of GBP. Under a CPP paradigm, male BALB/c mice were intraperitoneally injected either saline or 100, 200, or 300 mg/kg of GBP and confined to the injection-paired chamber for 30 min. In the pre-conditioning phase, mice were conditioned for 3 days, and baseline data were collected. In the conditioning phase, mice were given once-daily alternating injections of either GBP or saline for 8 days and subsequently assessed in a post-conditioning test. Injections of 300 mg/kg of GBP significantly increased the time spent in the drug-paired chamber compared to the saline-paired chamber. However, lower doses of GBP (100 and 200 mg/kg) showed no effect. Pre-treatment with SKF-83566, a D1 receptor antagonist, attenuated GBP-induced CPP. Thus, for the first time, we show that GBP can induce CPP through a dopaminergic-dependent mechanism.

## Introduction

The abuse and misuse of prescription drugs is a rapidly growing problem worldwide. The non-medical use of prescription drugs is universally considered one of the highest risk factors for poor health^1^. The abuse of gabapentinoids (i.e., pregabalin and gabapentin [GBP]), in particular, has been growing, and in 2016, gabapentinoids were one of the most commonly abused prescription medications globally^2,3^. Clinically, GBP is used as an adjunctive drug for treating partial epilepsy^4^ and for treating neuropathic pain associated with post-herpetic neuralgia^5^. Historically, GBP was assumed to have little or no potential for abuse, and this has contributed to dramatic growth in GBP prescriptions^2,6^. Also, GBP was proposed to aid in the management of cocaine dependence, benzodiazepine, and alcohol detoxification as it can protect from seizures induced by withdrawal symptoms^7^. Initial research proposed that GBP may be useful for treating drug and alcohol withdrawal symptoms, thus increasing the likelihood of gabapentinoid abuse among vulnerable patients^8^.

The mechanism underpinning gabapentinoid abuse has not yet been thoroughly investigated. Gabapentinoids are anticonvulsant drugs that can bind with high affinity to the alpha 2-delta1 (α2-δ1) subunit and with low affinity to α2-δ2 subunit but have no affinity for α2-δ3 subunits^9^. This binding is believed to result in blocking of calcium influx by voltage-gated channels^10^. Several studies have suggested that gabapentinoids can modulate the release of various neurotransmitters (for review see^11^). Of note, studies have found that most drugs that can modulate GABA possess abuse potential, such as alcohol and benzodiazepines^12^. Furthermore, it has been suggested that the dopaminergic system could be involved in the gabapentinoid abuse mechanism as gabapentinoids produce euphoria and withdrawal symptoms upon discontinuation^13^.

The administration of mind-altering drugs can impact the concentration and availability of numerous neurotransmitters in the central nervous system^14^. Dopamine, in particular, is a neurotransmitter that acts through the mesocorticolimbic dopamine system to facilitate reinforcing behaviors, and its regulation by drugs of abuse is strongly linked to addiction^15^. Indeed, studies in animal models have found that the reinforcing effects of different drugs of abuse such as methamphetamine, cocaine, and heroin are associated with high levels of dopamine in the mesocorticolimbic system^16^. Moreover, the release of dopamine in the mesocorticolimbic system is thought to be related to the activity of drugs of abuse in the ventral tegmental area, which projects into the nucleus accumbens and the medial prefrontal cortex, among other brain regions^17^. Repeated exposure to these drugs produces a behavioral sensitization state that ultimately promotes addiction^18^. Interestingly, blocking dopamine receptor activity prevented reinforcement behavior and behavioral sensitization associated with the repeated administration of most drugs of abuse, including methamphetamine, cocaine, and heroin^19–21^. Likewise, blocking the dopamine receptor attenuated heroin- and nicotine-seeking behavior in animal models of relapse^21,22^. Of note, D1 receptors, rather than D2, have been consistently shown to play a crucial role in mediating reward and its translation into action^23–27^. However, D2 receptors have been suggested to be involved more in mediating motor functions^28,29^.

In addressing the addictiveness of GBP, controversial data suggested that in rats administered GBP in a conditioned place preference (CPP) model, a maximum dose of 100 mg/kg GBP had no impact on CPP^30^. However, to date, no pre-clinical studies have investigated the addictive potential of higher doses of GBP or the involvement of the dopaminergic system in GBP-induced reward. Thus, in this study, we used a CPP mouse model to investigate the addictive potential of GBP and role of D1 receptors in GBP-induced reward.

## Results

### The effect of saline, or GBP (100, 200, and 300 mg/kg) on CPP

**Figure 1:**
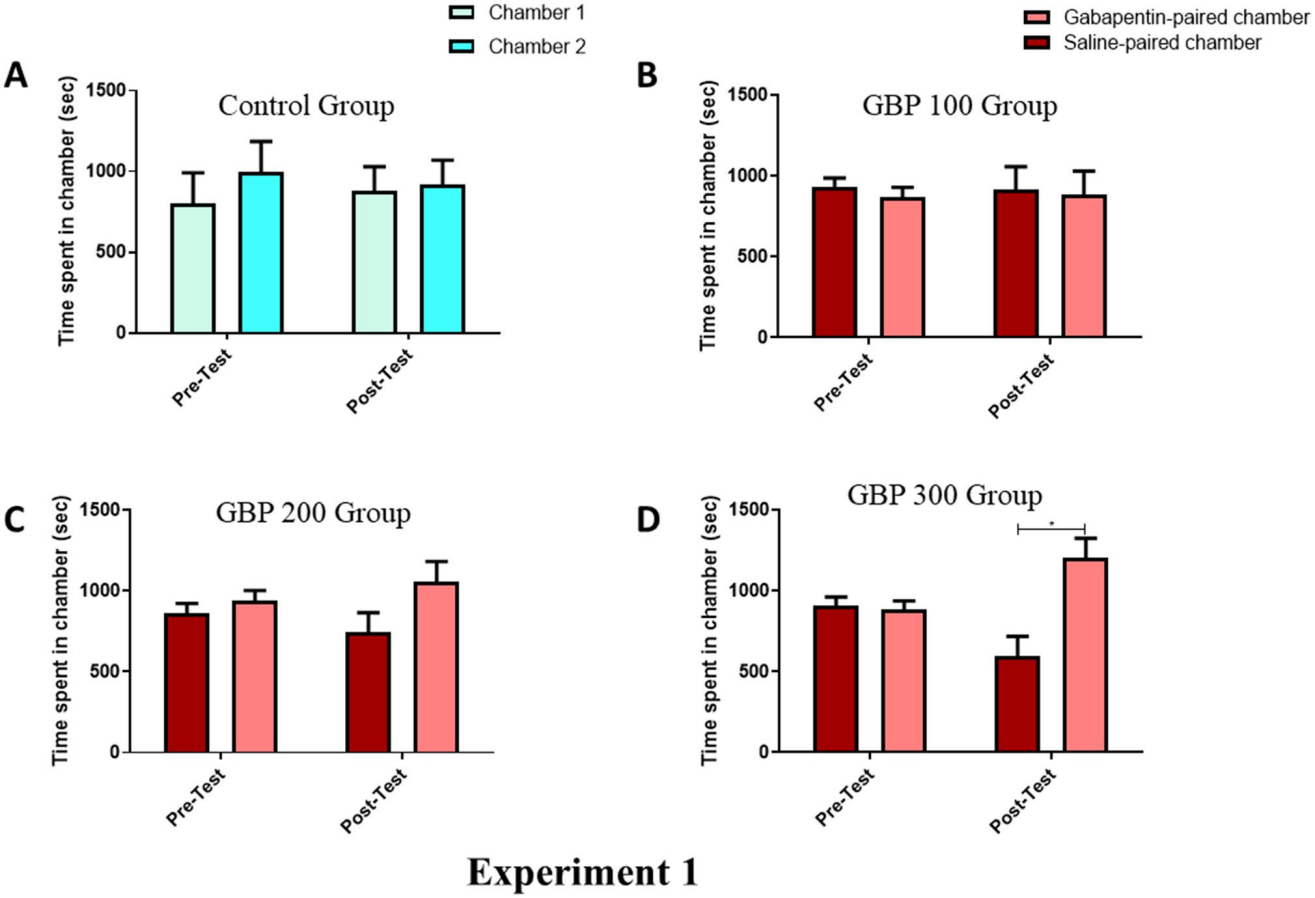
Time spent in conditioning chambers during phases of pre-test and post-test in the groups of the experiment 1: the control (**A**), GBP 100 (**B**), GBP 200 (**C**), and GBP 300 (**D**) group. No significant changes in time spent were found in chambers during all tested phases in the control, GBP 100 and GBP 200 groups. In GBP 300 group, a significant increase in time spent in GBP-paired chamber as compared to the Saline-paired chamber was found. *p < 0.05. Values shown as means ± S.E.M. N = 7–9/group.

In experiment 1, the potential for GBP to induce seeking behavior was tested using either saline or three different doses (100, 200, and 300 mg/kg) of GBP in mice. A two-way RM-ANOVA analysis was used to identify changes based on the phase, the preference for a chamber, or an interaction between the two. The saline only group (Fig. 1A) helped to assess the presence of confounding effects present within the injection or CPP procedures, which could impact the animals’ behavior. Importantly, the saline group showed no significant differences with respect to the phase [F (1, 6) = 1.000, *p* = 0.3559], chamber [F (1, 6) = 0.0006571, *p* = 0.9804], or an interaction between the phase and chamber [F (1, 6) = 0.002320, *p* = 0.9631].

Injecting mice with 100 mg/kg of GBP (Fig. 1B) did not show a significant impact on the phase [F (1, 8) = 0.7852, *p* = 0.4014], the preference for a chamber [F (1, 8) = 0.07087, *p* = 0.7968] or an interaction between the phase and the chamber [F (1, 8) = 0.009858, *p* = 0.9234]. The injection of 200 mg/kg GBP (Fig. 1C) resulted in a significant difference in the phase [F (1, 7) = 6.162, *p* = 0.0421] and no significant difference with respect to the chamber [F (1, 7) = 1.681, *p* = 0.2359] or between the phase and the chamber [F (1, 7) = 0.8885, *p* = 0.3773]. Moreover, Newman-Keuls multiple comparisons test did not show any significant difference in time spent between the GBP- and saline-paired chambers in pre- or post-test. We did not find any significant changes in time spent in the GBP-paired chamber during pre-test compared to post-test. Similarly, no significant changes were found in time spent in the saline-paired chamber during pre-test compared to post-test. Finally, injections of 300 mg/kg GBP (Fig. 1D) showed no significant difference with respect to the phase [F (1, 7) = 3.530, *p* = 0.1023] and chamber [F (1, 7) = 3.186, *p* = 0.1174]; however, there was a significant interaction between the phase and chamber [F (1, 7) = 11.37, *p* = 0.0119]. Moreover, Newman-Keuls multiple comparisons test showed a significant increase in time spent in the GBP-paired chamber compared to the saline-paired chamber during post-test (*p* < 0.05).

### Assessing the effect of D1-receptor antagonist on GBP-induced CPP

**Figure 2:**
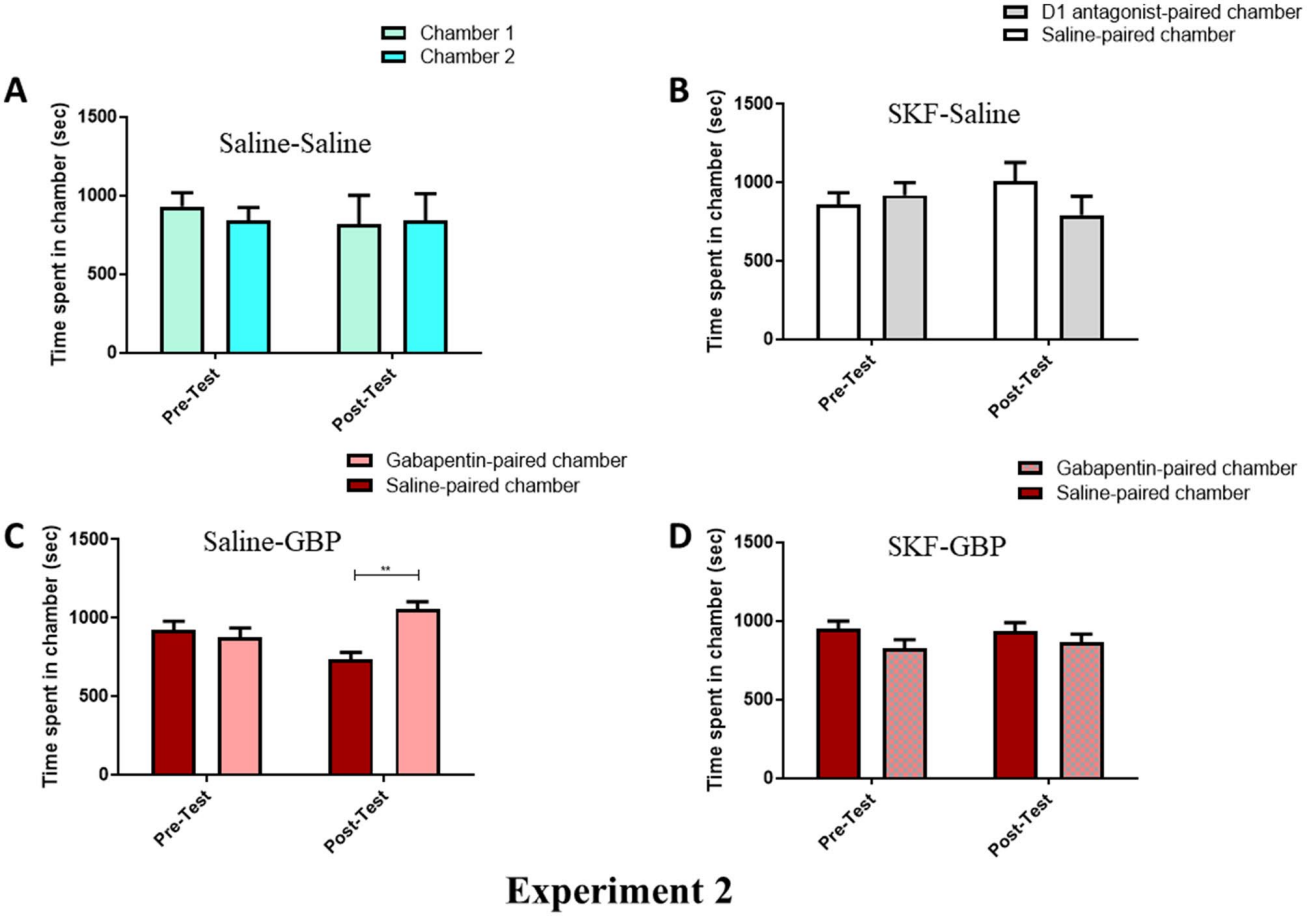
Time spent in conditioning chambers during phases of pre-test and post-test in the groups of the experiment 2: the saline-saline (**A**), SKF-S (**B**), S-GBP (**C**), and SKF-GBP (**D**) group. No significant changes in time spent were found in chambers during all tested phases in the Saline-Saline and SKF-S groups. In the S-GBP group, a significant increase in time spent in the GBP-paired chamber as compared to the saline-paired chamber was found. SKF pretreatment blocked the GBP-induced increase in time spent. **p < 0.01. Values shown as means ± S.E.M. N = 6–8/group.

In experiment 2, the ability for a D1-receptor antagonist to block the effect of GBP on inducing CPP was assessed. A two-way RM-ANOVA showed that saline-saline mice (Fig. 2A) were not significantly impacted by the phase [F (1, 5) = 0.1542, *p* = 0.7108], the chamber [F (1, 5) = 0.05726, *p* = 0.8204], or an interaction between phase and chamber [F (1, 5) = 0.1523, *p* = 0.7124]. Similarly, mice pre-treated with SKF-83566, a D1 antagonist, 30 min before saline post-treatment (Fig. 2B) were not significantly impacted by the phase [F (1, 5) = 2.490, *p* = 0.1754], chamber [F (1, 5) = 0.2304, *p* = 0.6515], or by an interaction of the phase and chamber [F (1, 5) = 1.281, *p* = 0.3091]. When saline was injected as a pretreatment and followed by a GBP (300 mg/kg) posttreatment (Fig. 2C), the phase [F (1, 6) = 2.358, *p* = 0.1756] and chamber [F (1, 6) = 2.264, *p* = 0.1831] had no effect. However, as in experiment 1, there was a significant interaction between the phase and chamber [F (1, 6) = 16.55, *p* = 0.0066]. As revealed by Newman–Keuls multiple comparisons test, saline-GBP mice spent more time in the GBP-paired chamber than in the saline-paired chamber in the post-test (*p* < 0.01). Interestingly, when SKF-83566 was given as a pretreatment, mice injected with GBP 300 mg/kg 30 min later displayed no phase effect [F (1, 6) = 1.001, *p* = 0.3556], chamber effect [F (1, 6) = 2.156, *p* = 0.1924], or phase and chamber interaction [F (1, 6) = 0.1071, *p* = 0.7546] (Fig. 2D).

## Discussion

In this study, for the first time, we demonstrated that GBP can induce drug-seeking behavior in an animal model of drug addiction. Moreover, we showed that blocking D1 receptor activity could prevent GBP-induced CPP. Our results are in accordance with those of several reports on the potential misuse, abuse, and withdrawal associated with GBP^12,31–34^. The number of gabapentinoid misuse, abuse, dependence, and overdose incidences reported by the Food And Drug Administration Adverse Events Reporting System (FAERS) between 2012 and 2016 is alarming and requires immediate attention from the regulatory authorities^2^. Similarly, in Europe, the adverse drug reactions (ADRs) system provided by The European Medicines Agency (EMA) reported that 4,301 cases from 90,166 GBP recipients between 2004 and 2015 had abused, misused, or were dependent on GBP^35^. Similarly, according to the results of the ADRs reporting system in Sweden, pregabalin has been concluded to have a potential for abuse^36^.

Recent reports indicate that GBP abuse commonly occurred when it was used recreationally, combined with other addictive drugs, synergized with existing prescriptions, used to control mood or anxiety, used as a substitution for other drugs, or used in drug abuse treatment^31,37^. In addition, several cases have reported that high doses of GBP can cause dependency and drug intoxication, which in turn is associated with delirium^12,38, 39^. These cases raise an alarming concern about the off-label prescription of GBP to patients with psychiatric conditions. The most common symptoms associated with GBP use include relaxation, delirium, euphoria, talkativeness, increased energy and dissociation, sedation and cognitive difficulties, and other side effects while taking the medication^6,40–42^. Many of these side effects commonly occur with other addictive substances.

The CPP procedure is widely accepted for studying and evaluating motivational properties, including the rewarding effects of drugs of abuse in experimental animals^43–47^. Under the CPP paradigm, we used a range of GBP doses (100, 200, 300 mg/kg) higher than those used by previous studies that used doses of GBP such as 10, 30, and 100 mg/kg and did not elicit any increase in place preference in rats^30,48^. Consistent with this previous work, in our analysis, 100 mg/kg of GBP did not result in place preference. Of note, there was a trend of increase in time spent in the GBP-paired chamber following the 200 mg/kg dose administration, but the effect did not reach statistical significance. Interestingly, only 300 mg/kg significantly increased the time spent by mice in the drug paired chamber during the posttest. This indicates that a high dose of GBP can induce CPP. This is consistent with our previous findings that the newer class member, pregabalin, can induce CPP, suggesting its possible abuse potential^49^.

Although the mechanism of GBP abuse is not completely understood, given that alcohol, nicotine, and opiates have high abuse potential and modulate dopamine, it is thought that the GBP mechanism is also dopamine-mediated^50^. The release of dopamine in the nucleus accumbens and subsequent activation of D1 receptors have been consistently shown to cause rewarding effects^51,52^. We showed that pre-treatment with a D1 receptor antagonist prevented GBP-induced CPP. This suggests that GBP-induced CPP, and by extension GBP dependence, is linked to the activation of D1 receptors.

In line with our findings, commonly reported motivations for GBP use among abusers include recreation (i.e., to get high), self-medication to address pain, self-harm, or to treat withdrawal symptoms from other substances. In many of the previous case reports, most individuals used GBP at doses higher than the prescribed dose. Descriptive reports indicate that GBP induces subjective effects, including euphoria, talkativeness, increased energy, and sedation. These effects are not specific to a particular dose and might occur within the therapeutic dose^32^. Moreover, the dose taken did not correlate with any effect or with the motivation for its use. This might be due to non-linear bioavailability of GBP and its unpredictable pharmacokinetics. Further experimental research is needed to characterize and evaluate GBP psychopharmacology and to uncover the risks associated with GBP use, especially among those who use it for recreational purposes^32^.

To the best of our knowledge, this is the first pre-clinical study that has explored the ability of GBP to cause rewarding effects. Furthermore, we showed that the suppression of D1-receptor activation can suppress GBP-induced place preference, indicating a critical role for dopamine in facilitating the addictive tendencies of GBP. Therefore, there is a need to impose further regulations to restrict the prescription of GBP. Moreover, additional research is needed to re-evaluate the potential for GBP to be abused and misused, especially in patients with a history of drug addiction.

## Methods

### Animals

Male BALB/c mice aged 6–8 weeks and weighing 25–35 g were obtained from King Fahad Medical Research Center (KFMRC, Jeddah, SA) and acclimatized in plastic cages for one week, during which animals were gently handled once per day to minimize stress. Mice were housed in groups of 5 per cage with ad libitum feeding. Isolated housing was avoided because it has been shown to affect the reinforcing behavior of different drugs of abuse^53–56^. Mice were housed at a constant temperature of approximately 21 °C and a humidity of 30%, under a 12-h light/dark cycle; all experiments including CPP were performed during the light cycle. All animal experiments were approved by the Research Ethics Committee at Taif University and conducted in accordance with the Institutional Animal Care and Use Committee of the National Institutes of Health guide.

### Drugs

GBP was obtained from Riyadh Pharma (Riyadh, SA). 8-bromo-2,3,4,5-tetrahydro-3-methyl-5-phenyl-1H-3-benzazepin-7-ol (SKF-83566) was obtained from Tocris Bioscience (MO, USA). Both drugs were dissolved in (0.9 NaCl) saline (vehicle).

### Experimental design and dose selection

*In experiment 1,* mice were randomly assigned to receive either 100, 200, 300 mg/kg of GBP or 10 ml/kg of saline (vehicle). The selection of doses of GBP was based on a previous study, which showed that doses of 100–300 mg/kg when given parenterally produced both CPP and antinociception in rodents^57–59^.

*In experiment 2,* mice received a pretreatment of 0.03 mg/kg of a D1 receptor antagonist (SKF-83566) or 10 ml/kg of saline (vehicle) 30 min before the injection of 300 mg/kg of GBP. It has been previously shown that 0.03 mg/kg SKF administered i.p. can attenuate the effects of d-amphetamine when tested in a free-operant psychophysical procedure^60^. Mice were handled gently to limit the potential for confounding effects on the behavioral testing of the mice.

### Conditioned place preference apparatus

The apparatus consisted of two acrylic chambers, identical in size (35 cm × 35 cm × 50 cm) and separated by removable walls. The chambers had different visual and tactile cues. One chamber had rough white walls with horizontal black stripes and round holes on the floor. The other chamber had smooth black walls with vertical white stripes walls and rectangular holes on the floor.

### Conditioned place preference procedure

#### Experiment 1

**Figure 3:**
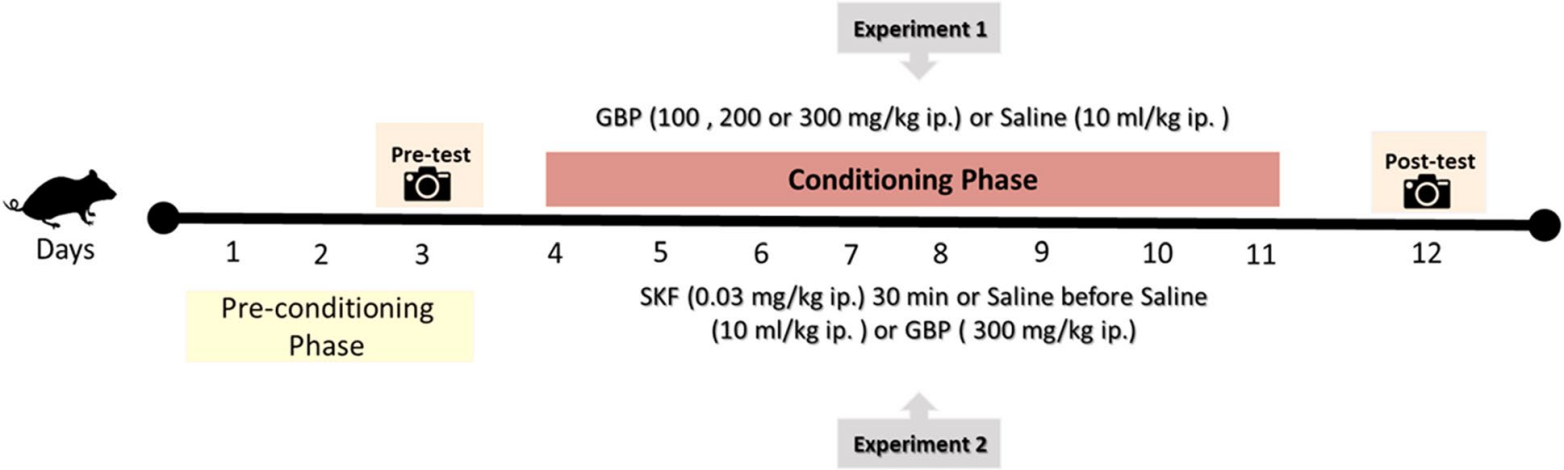
Experimental schedule of the CPP.

The involvement of the dopaminergic system in GBP reward was assessed using the CPP model. In this experiment, mice were randomly divided into four groups: a control group and 3 doses of GBP (100, 200, and 300 mg/kg). The CPP procedure was performed as described in our previous studies^46,49^. The experiments and drug dosages are illustrated in Fig. 3. Baseline preference was determined by placing each mouse in the start chamber of the CPP apparatus and allowing free access to the chambers for 30 min. This was done for the first three days (Day 1, 2 and 3). Each mouse underwent these three days of habituation to eliminate stress. On day three, the pre-test was conducted by placing the mice in the start chamber and allowing free access to both conditioning chambers for 30 min. The time spent in each chamber was recorded by automated processing using a digital camera and the ANY-maze video tracking software system. To ensure that there was no chamber preference bias, prior to conditioning test, we excluded any mouse who showed an initial preference to either chamber by spending > 67% of their time exploring that chamber ^46,61,62^. The four conditioning sessions using GBP were performed over an eight-day span with one session of GBP occurring every other day. Each mouse received only one injection per day of either GBP or saline. Mice received an injection of either saline, or 100 mg/kg, 200 mg/kg, or 300 mg/kg of GBP and were placed in one chamber for 30 min with the door closed. On saline treatment days, mice in all four groups received saline (10 ml/kg) and were subsequently placed in the other chamber for 30 min with the door closed. After completing the conditioning sessions, mice were tested for their chamber preference (post-test) on day 12. The post-test was identical to the pre-test procedure. The CPP design was un-biased and counterbalanced whereby half of the mice were randomly assigned to receive GBP in chamber 1 and the other half received this drug in chamber 2. Moreover, half of the mice were randomly divided to be administered GBP in the first conditioning day, while the other half received saline in the first conditioning day.

#### Experiment 2

The ability of dopamine D1 receptor antagonist SKF-83566 to block GBP-induced seeking like effect was tested. The baseline preference was assessed according to experiment 1. After completing the pre-test, mice were divided into four groups, with each group given two injections of a combination of either saline, SKF, or GBF. This included the saline-saline group, the SKF-saline group, the saline-GBP group, and the SKF-GBP group. Depending on their group, mice were first given an injection of either saline (10 ml/kg) or SKF (0.03 mg/kg). Mice were returned to their home cages for 30 min, and then given a second injection of either saline (10 ml/kg) or GBF (300 mg/kg) and subsequently placed in one of the separated chambers. On saline treatment days, mice in each of the four groups received saline (10 ml/kg) as pretreatment and saline as a posttreatment and were subsequently placed in the other chamber for 30 min, with the separating door closed. Mice were then tested for place preference (post-test) on day 12 after the completion of the conditioning sessions.

### Statistical analysis

Time spent in each chamber for pre-conditioning and post-conditioning was analyzed using a two-way repeated measures analysis of variance test (RM-ANOVA) (Phase x Chamber). Further post-hoc testing was performed using the Newman-Keuls multiple comparisons test. All analysis was conducted using GraphPad Prism (version 5.0; GraphPad Software). A p-value < 0.05 was considered statistically significant.
